# Syphilis-Related Eye Disease Presenting as Bilateral Papilledema, Retinal Nerve Fiber Layer Hemorrhage, and Anterior Uveitis in a Penicillin-Allergic Patient

**DOI:** 10.1155/2018/2840241

**Published:** 2018-02-18

**Authors:** Jamie Dietze, Shane Havens

**Affiliations:** ^1^University of Nebraska Medical Center, S. 42nd and Emile St., Omaha, NE, USA; ^2^Truhlsen Eye Institute, 3902 Leavenworth St., Omaha, NE, USA

## Abstract

*Purpose*. *Treponema pallidum* is known as the “great masquerader” for its many presentations and ocular findings in patients who are infected and develop secondary and tertiary stage of syphilis. Syphilitic ocular manifestations include uveitis, chorioretinitis, retinitis, vasculitis, vitritis, and panuveitis all with or without decreased visual acuity. Human immunodeficiency virus (HIV) is known to expedite the progression of syphilis when patients are coinfected, thus compounding the potential ophthalmic presentations. This report summarizes the presentation, management, and clinical course of a patient with known HIV and penicillin allergy that presented with bilateral optic nerve edema, retinal hemorrhages, and iritis without vision loss.

## 1. Introduction

Neurosyphilis is a known complication from infection with *Treponema pallidum* that has many different types of ocular manifestations [1]. Patients who progress to neurosyphilis typically have anterior uveitis unless there is coinfection with HIV, which appears to facilitate an involvement of the posterior segment [2, 3]. Ocular symptoms can still occur in the setting of good compliance with highly active antiretroviral therapy (HAART), but posterior uveitis is more common when CD4 counts are <200 [4]. Ocular manifestations seen in HIV-associated neurosyphilis include posterior uveitis, panuveitis, posterior placoid chorioretinitis [5], retinal vasculitis, and, on rare occasions, bilateral optic neuritis [3, 6]. However, most of these presentations have an associated decrease in visual acuity [7]. The established treatment for cases of ocular syphilis is to treat the systemic infection with Penicillin G which can result in improvement of the ocular changes, including improvement of uveitic symptoms such as photophobia and visual acuity loss [3, 4, 7]. When patients have a history of immediate-type hypersensitivity reactions to penicillin, they must be desensitized prior to starting treatment to allow short-term tolerance [8]. This desensitization can be done either orally or IV but must be done in a hospital setting due to the potential risk of IgE-mediated allergic reactions.

## 2. Case

A 50-year-old Caucasian male with a history of multiple drug-resistant HIV but without prior retinopathy presented with complaints of seeing strobe-like photopsia in both eyes, constant tearing from the left eye for 2 weeks with pain nasally when rubbed, chronic photophobia, and black/white temporal flashes bilaterally for 1 week but no decreased vision. The rest of his review of symptoms was benign with pertinent negatives for recent fever, illness, malaise, headache, neck stiffness, rash, or genital lesions.

He has a social history pertinent for multiple prior male sexual partners, one of which was known to also be HIV positive. He reported the use of condoms for protection and is a current smoker. He has no recent travel history except to a state fair. His past medical history is significant for CKD stage II and asthma with allergies to penicillin and aspirin.

**Figure 1:**
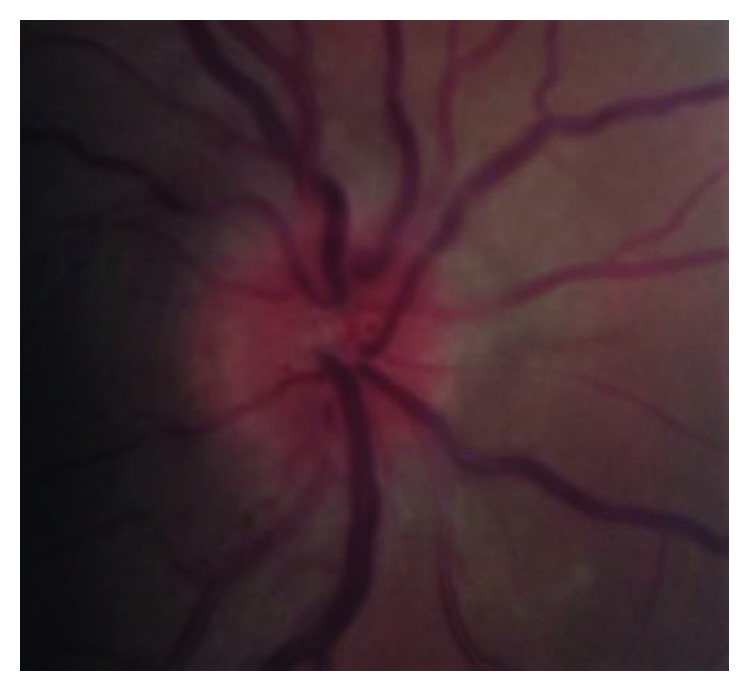
Right eye optic nerve head color photo shows a circumferential halo around optic nerve consistent with mild-moderate optic nerve head edema with a cup-to-disc ratio of 0.1 and retinal venous congestion.

**Figure 2:**
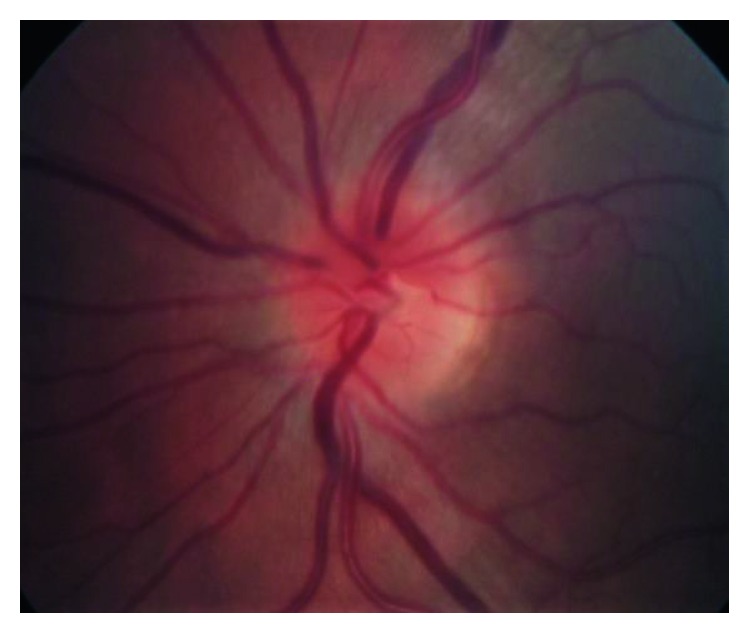
Left eye optic nerve head color photo showing a C-shaped halo with temporal (right) gap around the optic nerve consistent with mild optic nerve head edema with a cup-to-disc ratio of 0.1.

On exam, visual acuities were 20/25 in right eye (OD) and 20/20 in the left eye (OS) with correction, normal brisk pupillary reaction, intact extraocular muscle movements, and a normal anterior chamber eye exam in both eyes (OU). Eye pressures taken were 13 mmHg OD and 11 mmHg OS and a cup-to-disc ratio of less than 0.1 bilaterally (OU) as shown in Figures 1 and 2.

**Figure 3:**
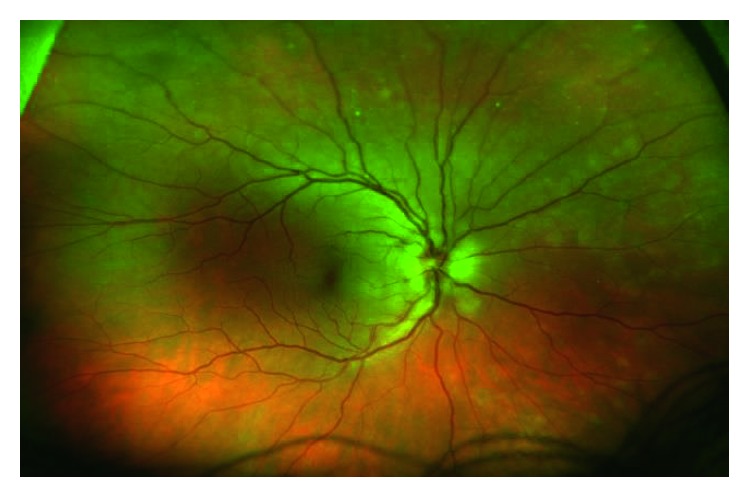
Right eye wide-field color photo demonstrating retinal nerve fiber layer hemorrhage temporally (left). Patchy plaques in the choroid are evident in the nasal (right) and superior midperiphery.

**Figure 4 fig4:**
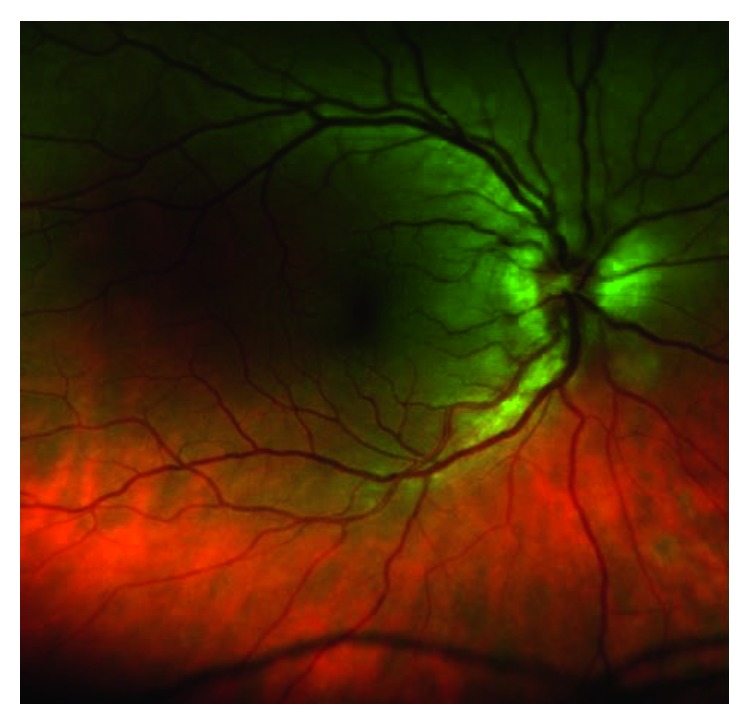
Right eye posterior pole color photo of retinal nerve fiber layer hemorrhage and patchy changes in the choroid.

**Figure 5 fig5:**
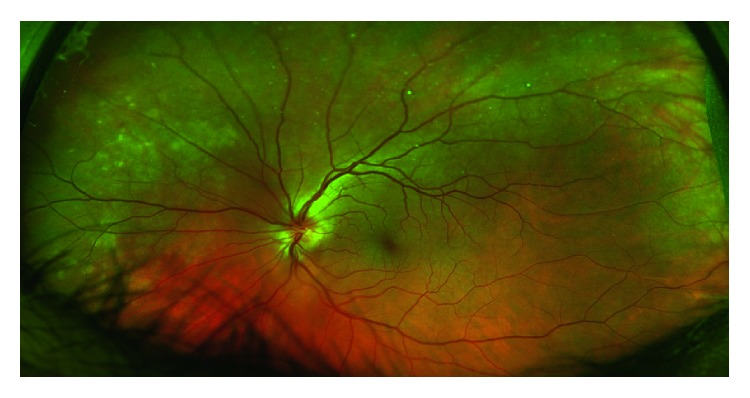
Left eye wide-field color photo demonstrating similar pale plaques in the choroid superiorly and nasally (left).

**Figure 6 fig6:**
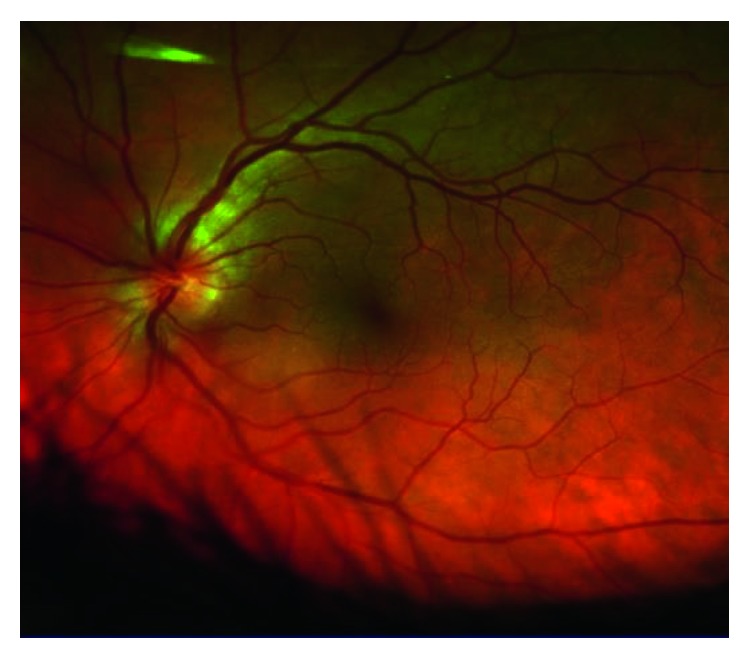
Left eye posterior pole color photo showing mild venous congestion.

**Figure 7:**
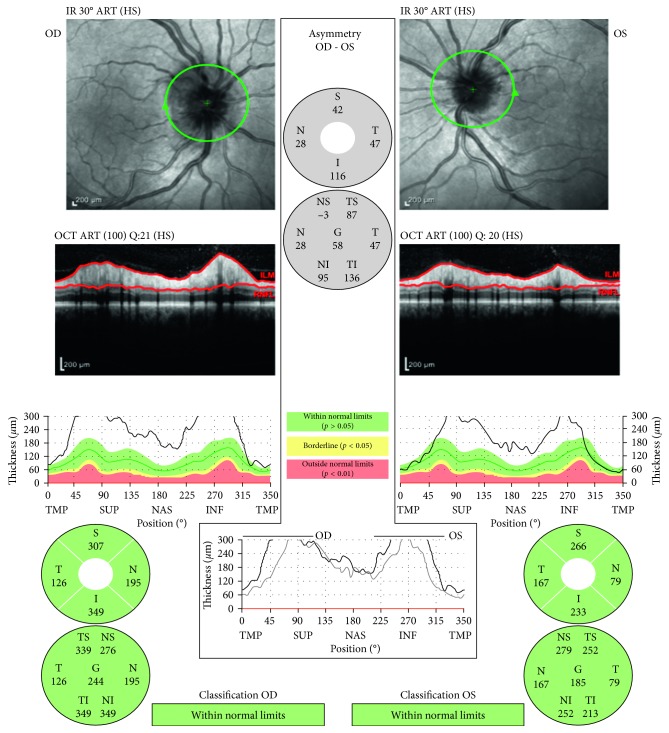
Optic nerve head optical coherence tomography showing significant optic nerve head edema bilaterally. The right eye optic nerve head shows slightly greater edema than the left eye, consistent with bilateral optic nerve head edema.

Dilated funduscopic examination revealed mild-moderate optic nerve head edema with small temporal nerve fiber layer hemorrhage in the right eye and mild optic nerve head edema in the left eye as shown in Figures 3 [Fig fig4] [Fig fig5] [Fig fig6]–7.

**Figure 8:**
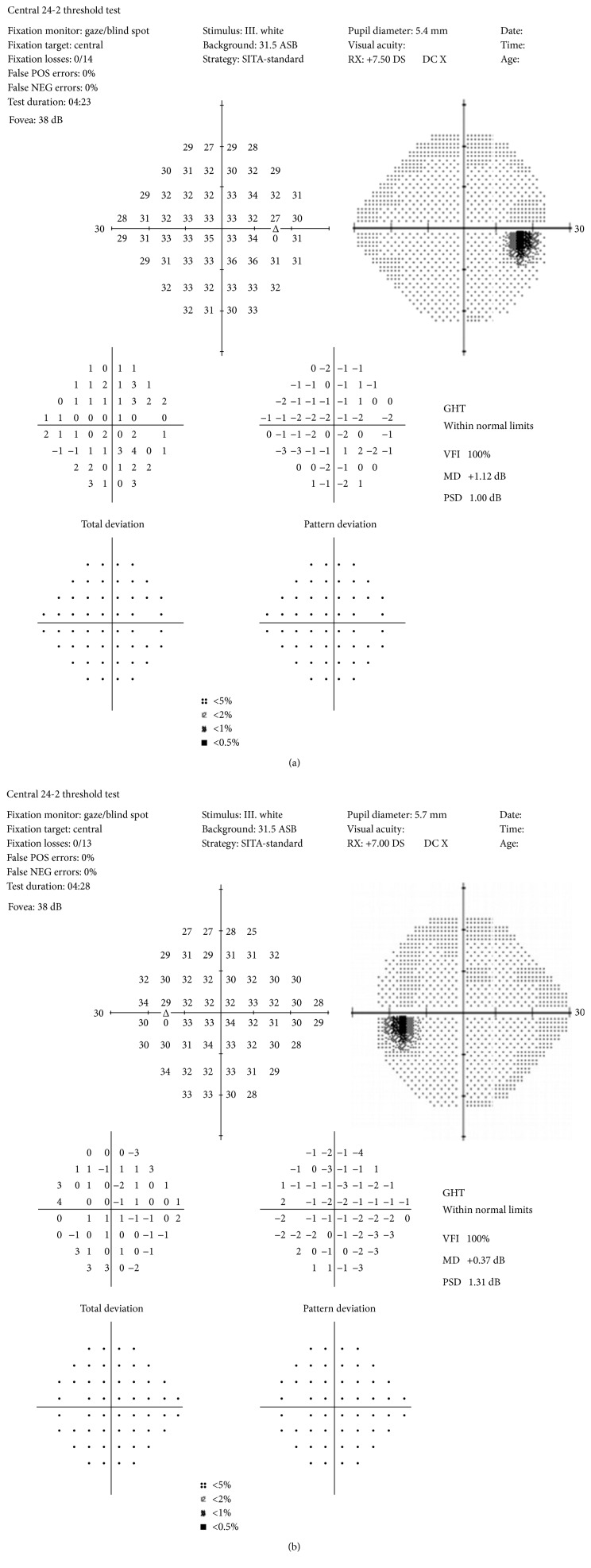
Humphrey visual fields were full and reliable in both eyes. The points of decreased vision shown are consistent with the normal physiologic blind spot caused by the optic nerve.

He denied headache or focal neurodeficits as well as no visual field deficits. Full visual fields were confirmed by Humphrey visual field testing as shown in Figure 8.

Blood levels showed a normal CD4 count and an undetectable viral load, making an opportunistic infection less likely. The patient was sent for MRI and lumbar puncture with CSF evaluation.

MRI was remarkable for mild nonspecific white matter changes, prominent leptomeningeal enhancement throughout cranium with a normal orbit. No masses, midline shift, or hydrocephalus was present. Lumbar puncture revealed CSF with 18% PMNs but was negative for VDRL. Blood samples showed reactive rapid plasma regain (RPR) with a 1 : 128 titer and positive IgG *Treponema pallidum* antibody (TPA). CSF was also tested for cryptoantigen, AFB, West Nile, *Borrelia burgdorferi*, CMV, HSV, HHV6, VZV, EBV, and fungal culture, which all returned negative except for an equivocal level of 1.00 antibody to *Borrelia burgdorferi*.

Patient was admitted to the ICU and desensitized to IV Penicillin G via PICC line. He was given a pretreatment dose of hydrocortisone due to his asthma and smoking history, and then penicillin G doses were titrated up by a power of 10 starting at 0.4 units until a dose of 4 million units for a total of 24 million units q24h. He was then discharged to home dose of 4 million units IV q4h for 14 days. The patient was given an epinephrine pen and Benadryl PRN. The patient did develop a nonpruritic, nonpainful mild rash during the desensitization course, but the patient tolerated it well and could be attributed to a Jarisch–Herxheimer reaction.

One week after initial presentation, the patient was evaluated in follow-up. Exam revealed stable optic disc edema, new cells with trace flare in the anterior chamber, and a round, reactive iris without other symptoms of iritis. The rest of the eye exam was normal, and the patient had a review of systems only pertinent for still having a stable rash on the abdomen at this time.

After finishing the IV Penicillin G regimen and continual use of HAART, optic nerve edema completely resolved after 3 months. At this time, his TPA was still positive as expected and his RPR was 1 : 4 which was then checked every 4 months until results became nonreactive. After 13 months from initial presentation, his RPR titer was nonreactive, confirming a successful treatment.

## 3. Discussion

In this case, there were a unique set of diagnostic and treatment challenges posed. Visual scintillations, unilateral optic pain, and bilateral optic nerve edema with a retinal nerve fiber layer (NFL) hemorrhage were the first manifestations of neurosyphilis in an HIV coinfected patient. While there are a couple reports of bilateral papillitis being one of the findings on initial presentation, they were associated with a more robust unilateral focal chorioretinitis or a chorioretinitis with decreased visual acuity [3]. This is the first reported case with NFL hemorrhage and thickening and iritis concurrent with papillitis without decreased visual acuity or visual field loss. Due to this atypical presentation and the patient's coinfection of HIV, emergent and opportunistic diagnoses needed to be ruled out first with MRI and a CD4 count.

Syphilis and HIV have been well documented to coincide together especially in other countries with higher endemic rates. Rates of coinfection as high as two-thirds in HIV infected people are reported in one study [9]. Coinfection with HIV puts patients at an increased risk of neurological complication, ophthalmic involvement, and treatment failure, often presenting as an aggressive secondary stage disease [2, 9, 10].

The CDC guidelines recommend CSF examination with CSF-VDRL or CSF FTA-abs for patients with neurologic or ophthalmic signs and symptoms with HIV coinfection in order to confirm a diagnosis of syphilis [11]. Without treatment, patients tend to have a more severe ocular inflammation and faster rate of progression than immunocompetent counterparts [9, 12].

Mainstay treatment of syphilis is Penicillin G [11]. Patients coinfected with HIV have an increased chance of developing Jarisch–Herxheimer reaction [9]. Patients with penicillin allergy who are not coinfected with HIV can receive alternative therapy. Such therapy options include 100 mg PO BID doxycycline for 28 days or ceftriaxone 2 g IV or IM daily for 10–14 days if desensitization to penicillin is not possible, but there are limited data for the efficacy of these alternative therapies when there is evidence of neurosyphilis [13]. Patients with known HIV and syphilis have not been studied with alternative therapy and are thus recommended to receive penicillin if desensitization can safely be completed.

Penicillin desensitization only allows for temporary tolerance of treatment; once the patient finishes treatment, the hypersensitivity returns. Patients should be selected for this therapy when they have proven by skin testing/drug-specific IgE testing or are strongly suspected to have immediate-type allergy based on clinical history, and there are no other acceptable therapy options. The general guidelines for IV penicillin desensitization are to double the dose at each step, starting with an initial dose that is 1/10,000 of the desired full dose over 15 minutes of continuous infusion. If no adverse reaction is observed, then proceed immediately to the next dosage step. Patients should stay on their beta-blockers if the risk of taking them off is high, and patients with asthma or lung disease should be pretreated with oral glucocorticoids in order to minimize airway inflammation [13]. Once titration to full dosage has been achieved, patients can be started on their treatment regimen via an IV or oral route in order to treat the syphilis infection.

## 4. Conclusion

In conclusion, this case presents a relatively uncommon combination of presenting findings: bilateral papilledema, nerve fiber layer hemorrhage and thickening, and iritis in a patient with HIV and syphilis. This case demonstrates how a patient with HIV, ocular syphilis, asthma, and a penicillin allergy can be safely and effectively managed with penicillin desensitization followed by a 14-day course of IV Penicillin G.
